# Organometallic Compounds and Metal Complexes in Current and Future Treatments of Inflammatory Bowel Disease and Colorectal Cancer—a Critical Review

**DOI:** 10.3390/biom9090398

**Published:** 2019-08-22

**Authors:** Adrian Szczepaniak, Jakub Fichna

**Affiliations:** Department of Biochemistry, Faculty of Medicine, Medical University of Lodz, 92-215 Lodz, Poland

**Keywords:** inflammatory bowel diseases, organometallic compounds, metal complexes, free radicals, colorectal cancer

## Abstract

In recent years, there has been a significant increase in the clinical use of organometallic compounds and metal complexes for therapeutic purposes including treatment of inflammatory bowel diseases (IBD). Their action is based on the inhibition of the inflow of pro-inflammatory cytokines, the elimination of free radicals or the modulation of intestinal microbiota. In addition, these compounds are intended for use in the diagnosis and treatment of colorectal cancer (CRC) which is often a consequence of IBD. The aim of this study is to critically discuss recent findings on the use of organometallic compounds and metal complexes in the treatment of IBD and CRC and suggest future trends in drug design.

## 1. Introduction

Inflammation is a natural reaction of the body in response to tissue damage, caused mainly by chemical and physical factors [1]. In the long-term it can lead to cancer development through genotoxic processes or improper tissue repair [2]. Intestinal inflammation mediated by the immune system is the most important feature in the development of inflammatory bowel diseases (IBD) [3]. It can be triggered by stimulation with lipopolysaccharide (LPS), tumor necrosis factor-alpha (TNF-α), interleukin-1 (IL-1) or interferon-gamma (IFN-γ). After initiation of the inflammatory process, the production of cytokines and other mediators of inflammation increases through activated macrophages [4]. Consequently, molecules secreted by macrophages such as nitric oxide (NO) contribute to intensification of the inflammatory process [5]. The main intracellular pathways associated with the development of inflammation include transcription factors signal transducer and activator of transcription 3 (STAT3) and nuclear factor-κB (NF-κB) [2].

Inflammatory bowel diseases are most commonly represented by Crohn’s disease (CD), which covers any part of the gastrointestinal tract and ulcerative colitis (UC) that occurs in the large intestine or rectum [6]. The prevalence of IBD remains high in America and Europe but also has become more common in areas with previously low incidence, such as China and South America [7]. In Europe, the prevalence of UC is the highest, amounting to 24.3 per 100,000 people, while North America is the place where CD is mostly recognized with a frequency of 20.2 per 100,000 people [8,9]. Inflammatory Bowel Diseases strongly affect the patients’ quality of life through recurring symptoms such as abdominal pain, vomiting or diarrhea; existing therapies allow to reduce these symptoms but do not affect the propagation of the disease [10]. What additionally hampers the development of new efficient therapies in IBD is the fact that its etiology is not fully understood [11]. There are several factors that can contribute to development of IBD, which include immune system disorders, genetic changes or microbiome composition. It has also been shown that lifestyle has an influence on the occurrence of IBD. Studies on nutritional status proved that higher consumption of milk protein or polyunsaturated fatty acids may increase the risk of IBD [12]; tobacco consumption increases the risk of Crohn’s disease [13].

Colorectal cancer (CRC) is one of the most common cancers worldwide. It is ranked 3rd among men and 2nd among women in terms of frequency of occurrence, while the estimated number of new cases in 2018 is more than 1.8 million worldwide [14]. Development of CRC is a complex, multifactorial process involving the interactions of environmental and genetic factors. According to the latest reports, there is also strong evidence that increased consumption of processed meat, alcoholic beverages, smoking, physical inactivity or obesity significantly contribute to increased probability of developing CRC [15].

Moreover, patients with long-term UC and CD are more likely to develop CRC than the general population. It should be noted that the IBD-related CRC represents approximately 2% of all CRC [16], however, the mortality resulting from CRC in patients with IBD ranges from 10% to 15% [17].

Currently, sulfasalazine, corticosteroids, immunosuppressive agents such as azathioprine and biological therapy represented by anti-TNFα antibodies are used in IBD [18]. However, treatment with these agents produces adverse effects, requires long-term treatment periods and often presents a high rate of relapse, which limit their use [19].

Organometallic compounds and metal complexes are becoming more and more popular in medicine and their therapeutic properties are widely used in the treatment of several health disorders (for examples see Table 1). Organometallic compounds are defined as having one or more covalent metal-carbon bonds and a wide variety of structural diversity. In addition, they are characterized by kinetically stable, usually uncharged, and relatively lipophilic composition and therefore offer great opportunities for the design of new therapeutics [20]. Metal complexes consist of a central atom or ion (coordination center), which is metallic and bound molecules or ions. Organometallic compounds and metal complexes possess anti-inflammatory properties; they are also known to reduce the harmful effects of free radicals. In this review we will focus on their application for the treatment of inflammatory diseases of the digestive system.

## 2. Anti-Inflammatory Properties Related in IBD

### 2.1. Metal and Metal Complexes

#### 2.1.1. Zinc

**Figure 1:**
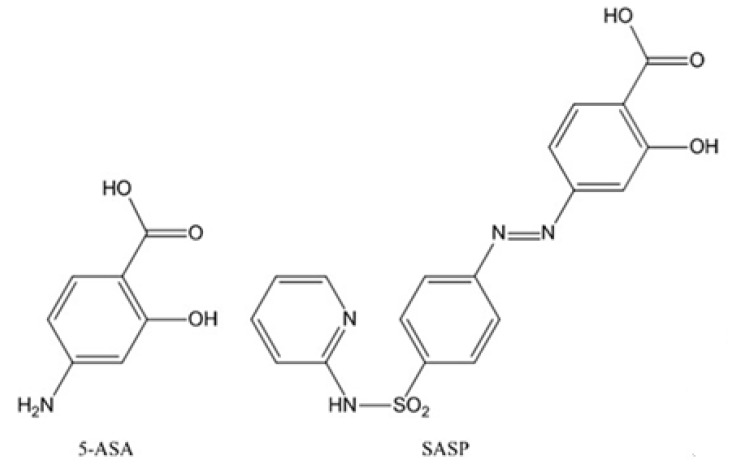
The structures of 5-aminosalicylic acid (5-ASA) and salicylazosulfapyridine (SASP) [28].

A large group of currently used non-steroidal anti-colitic compounds are all pro-drug formulations for 5-aminosalicylic acid (5-ASA). The major obstacle in IBD treatment with 5-ASA is its significant absorption from the gastrointestinal tract without reaching the colon [27]. An important alternative used in IBD therapy is salicylazosulfapyridine (SASP), which allows the transport of 5-ASA to regions affected by colitis, where the bacteria cleave the azo bond to release 5-ASA. However, this is not an ideal transport method because the carrier is absorbed through the intestinal wall causing toxic side effects (Figure 1) [28]. To improve the treatment, complexes with copper and zinc have been proposed, which both can serve as centers protecting the active drug from absorption in the stomach, allowing it to reach the colon. Moreover, zinc may additionally act as an effective remedy for damaged tissue; for example, animal studies showed that zinc protects against stomach ulceration [29]. Novel complexes containing salicylaldehyde (SA) and copper or zinc have been thus developed.

**Figure 2:**
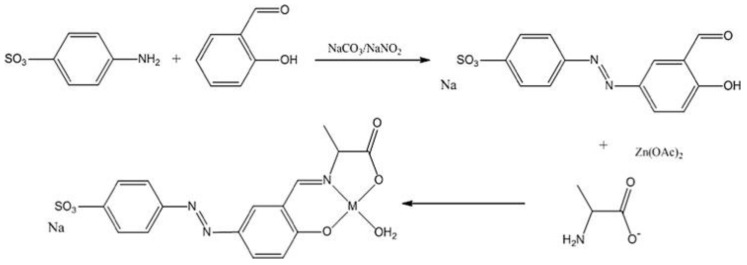
The synthesis of the trimodal zinc complex AzZnSA [28].

To test the action of the synthesized compounds, a mouse model of formalin induced colonic inflammation was used. It was observed that zinc performs better than copper in the studied complexes, yet the presence of alternative metal centers that could stabilize Schiff’s base bonds should be investigated. An important observation is the fact that oral administration of Zn-SA complexes significantly reduced the myeloperoxidase activity (58%) in the in vivo acute colitis model. The effect of another compound, sodium 5-(*p*-sulfophenylazo-)-salicylidenealanine-zinc (AzZnSA) on colonic inflammation was also investigated (Figure 2). The group treated with the highest dosage of AzZnSA demonstrated an absence of tissue adhesion or colonic distension. AzZnSA, like SASP, was effective in reducing the symptoms of colitis [28]. Myeloperoxidase is a lysosomal protein that is released to the neutrophil phagosome during the degranulation process, often becoming hyper-expressive in inflammatory diseases, including IBD; consequently, it is a good indicator of the process [30].

Patients with UC have a long-term watery or bloody diarrhea caused by collapse of rectal mucosa, therefore, one of the main clinical goals of UC treatment is an immediate repair of damaged or collapsed colon membrane. A zinc complex *N*-(3-aminopropionyl)-l-histidinato zinc is a drug used to treat peptic ulcers in Japan. However, animal studies have shown that it is effective against inflammation induced by subserosal injection of acetic acid in the ulcer healing phase [31]. Further studies on zinc-based organometallic complexes proved that a chelate compound that consists of zinc and l-carnosine (PZ) enema therapy is effective in patients with UC and that endoscopic and pathological findings were significantly improved after one week of PZ treatment [29]. In the same study, the concentration of zinc in the serum was measured at the beginning and after the 8th day of therapy and it was significantly higher on day 8 than at the beginning, but still in the normal range. Current results suggest that repairing damaged mucus membrane may become a new strategy for patients with UC. This strategy can also improve the quality of life of patients by reducing the number of bowel movements [29].

#### 2.1.2. Silver

Many studies indicate that silver nanoparticles (AgNPs) have exceptional anti-inflammatory properties in vitro, accelerating tissue repair and regeneration [32] and are thus employed in suture coating. Reports indicate that at the macroscopic level, sutures coated by AgNP caused reduced inflammatory response compared to “classical” suture coated with antibiotics and could contribute to “physiological” healing of intestinal tissue after injury [33]. Moreover, long-term action of AgNP-coated seams displayed better efficiency in healing of the intestinal tissues. It is speculated that the release of silver from the AgNP-stitched seam causes a significant reduction in local inflammation which contributes to faster and more effective tissue repair and regeneration. The positive effect on the inflammatory process may be due to the fact that the threads covered with the AgNP coating release pure silver in a prolonged manner, which leads to a strong anti-inflammatory effect. Moreover, it was found that AgNP inhibits the expression of pro-inflammatory cytokines. Namely, when threads with AgNP are used, lower concentrations of IL-6, IL-10 and TNF-α were found [33].

The use of anti-inflammatory properties of AgNP were also proposed in IBD. In a study with a rat model of UC, Bhol et al. [34] showed that NPI 32,101 (nanocrystalline silver) alleviates inflammation and reduces the production of IL-12, IL-1β, and TNF-α. In addition, NPI 32,101 decreases the activity of matrix metalloproteinase (MMP)-9 [34], elevated in patients with IBD, possibly following the infiltration of macrophages into the intestine [35].

Research indicates that other forms and formulations of silver, complexes included, induce apoptosis of inflammatory cells through oxidative stress, thus promoting the process of wound healing and alleviation of the inflammatory process in the gut. For example, studies carried out in the mouse model showed that silver-coated glass beads have anti-inflammatory effects on TNBS-induced colitis, which was mainly related to the presence of the silver nanolayer on the beads, and to a lesser extent to the size of the glass polymer units [36]. The obtained data confirm readiness to use this form of drug delivery in patients with IBD in a clinical setting.

Although previous studies indicated that silver-based compounds have anti-inflammatory effects and could be used in the treatment of inflammatory diseases, there is no sufficient research which could result in new products that may be used to treat IBD. The priority now is to focus on the research aiming at the development of new silver compounds and the determination of the mechanisms of their action.

#### 2.1.3. Gold

Gold(I)/(III) nanoparticles are used both in cancer [37] and antimicrobial [38] therapy. The key target of gold complexes are mitochondria and the selenoenzyme thioredoxin reductase (TrxR) [39]. In addition, gold complexes inhibit signal transducers and activators of transcription 3 (STAT3) or NF-κB [40], which indicates their potential use in the treatment of IBD. STAT3 is activated in response to cytokines and growth factors [41] and its role in the pathogenesis of IBD has been accurately described in recent studies. Moreover, it was shown that STAT3 is constitutively activated in CD patients, and a similar tendency was observed in patients with UC [42]. Gold compounds can also be used to inhibit NF-κB which has a beneficial effect on IBD because expression and activation of this transcription factor is induced in the inflamed gut of IBD patients and also is in the state of activation in mucosal macrophages [40,43].

Most of the studies on gold complexes in inflammation were principally focused on gold(I) complexes. Auranofin [2,3,4,6-tetra-o-acetyl-l-thio-β-d-glycopyranp-sato-*S*-(triethyl phosphine)-gold] is an approved treatment for rheumatoid arthritis, but has been studied for potential therapeutic use in many other diseases, including cancer and bacterial infections. The mechanism of action of auranofin is based on inhibition of reduction/oxidation (redox) enzymes such as TrxR. The inhibition of these enzymes leads to cellular oxidative stress and apoptosis [44].

**Figure 3:**
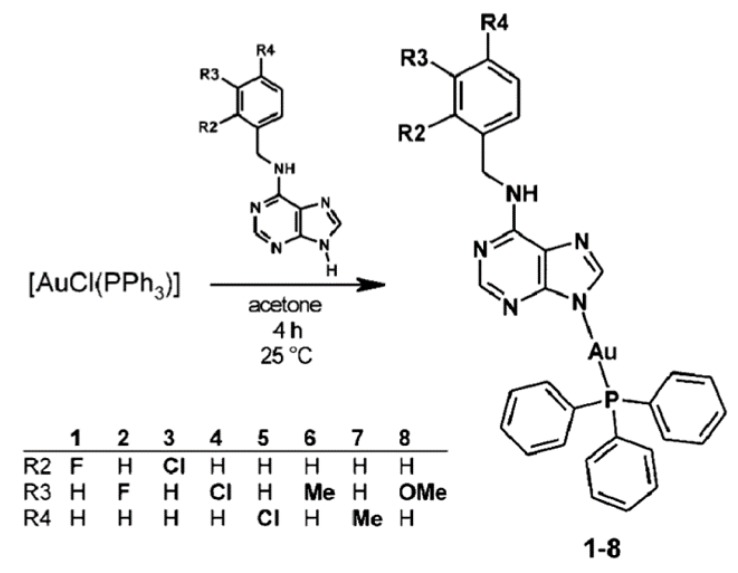
Synthetic pathway for the preparation of the gold(I) complexes (1–8) [45].

Travnicek et al. [45] succeeded in synthesis of a series of complexes (Figure 3) with anti-inflammatory activity, possibly due to their structural similarity to auranofin. Of note, complexes no. 1, 3 and 6 significantly decreased TNF-α secretion after LPS treatment, being two times more efficient than Auranofin. Moreover, complexes 1 and 3 significantly decreased the level of another pro-inflammatory cytokine, IL-1β. In addition, it was shown that complexes 1 and 6 significantly influenced the formation of edema caused by the application of polysaccharide λ-carrageenan in vivo. Importantly, all tested gold (I) complexes displayed lower cytotoxicity than Auranofin. Gold (I) complexes can become an effective treatment for inflammatory diseases. In the future, it is necessary to examine the precise mechanism of their action and their possible use as medicine [45].

The in vitro and in vivo studies that followed confirmed the anti-inflammatory properties of complexes designed by Travnicek et al. Interestingly, the time complexes 2 and 4 showed significant anti-inflammatory effects at both levels, comparable to the commercially used drug Auranofin. It was concluded that all complexes have a similar mechanism of action, but their effectiveness is likely to depend on bioavailability. The studies also indicated that the tested complexes reduce the production of proinflammatory cytokines due to blocking of the NF-κB signal pathway by inhibiting IkB degradation. This is in line with previous findings, in which Auranofin and other complexes containing gold are able to bind to the cysteine residues of IκB kinase (IKK) and thus block its function. Complexes 1–5 can thus be useful alternatives to cisplatin in cancer treatment as well as anti-inflammatory agents like Auranofin [46].

#### 2.1.4. Rhodium

**Figure 4:**
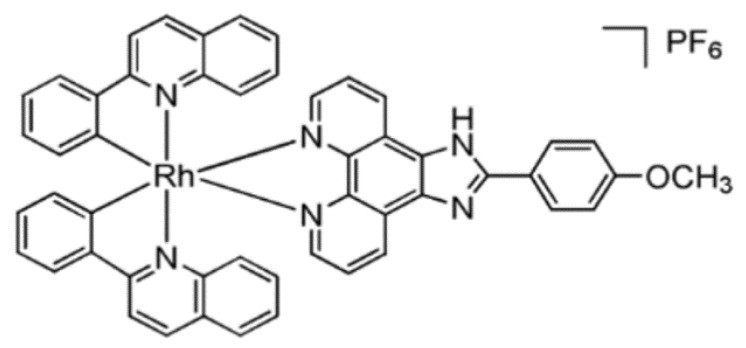
Chemical structure of rhodium(III) complex, a strong inhibitor of NEDD8-activating enzyme (NAE) [47].

The rhodium centers (III) have traditionally been considered kinetically inert, but it was shown that their reactivity to biological objectives could be increased by including appropriate ligands. Zhong et al. [47] developed a new Rh (III) complex, which they identified as an inhibitor of the NEDD8 activating enzyme (NAE) and confirmed its anti-inflammatory activity in the IBD mouse model (36). NAE is a ubiquitin-like E1 enzyme that is involved in the regulation of cullin/RING ubiquitin ligases (CRLs). NAE modulation can change the rate of ubiquitination and degradation of inflammatory proteins such as IκBα, p27 and NF-κB, which are strongly associated with IBD. Of note, it was proved that the presence of a methoxyl group in the final ligand position N∧N is important for NAE braking activity. Moreover, it has also been shown that the use of N-donor acetonitrile ligands can reduce NAE inhibition and the replacement of the Rh (III) center with Ir (III) (Figure 4) led to dramatically reduced NAE inhibitory activity [47].

### 2.2. Electron Deficient Organometallics

Ruthenium, osmium and iridium compounds are a versatile family of organometallics whose biological properties have raised expectations principally for the treatment of cancer [48,49]. However, the researchers are also focusing on their potential anti-inflammatory significance.

**Figure 5:**
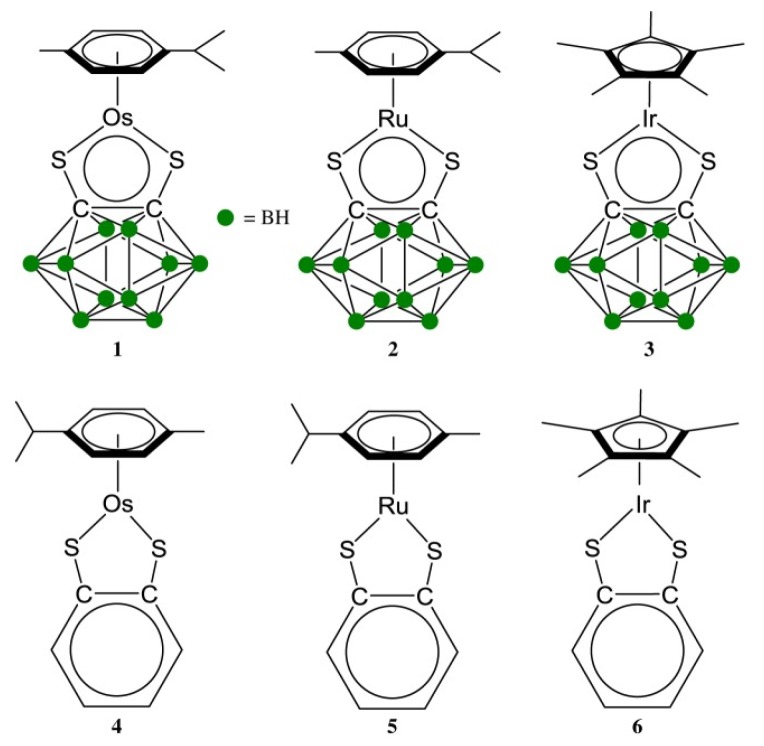
Molecular structures of the electron-deficient half-sandwich metal complexes studied in this work [4].

Zhang et al. [4] studied the activity of a series of electron deficient organometallic compounds (Figure 5) towards mouse macrophages RAW 264.7 and fibroblast cells MRC-5, which are widely used as in vitro models for inflammation. For this purpose, macrophage cells RAW 264.7 were treated with five different concentrations of tested compounds (0, 10, 20, 50 and 100 µM); all compounds slightly reduced cell viability after 24 h of treatment, except for compound 3 and 6, which did not induce any significant changes. The cytotoxic effects of compounds 1–5 were dose-dependent, where the main trend showed that with increasing concentrations, cell survival decreases. Compounds 1 and 2 showed concentration-dependent cytotoxicity while the activity of other metal complexes was concentration-independent. An important observation was that after 24-h recovery of cells, the compounds did not show any cytotoxicity to RAW 264.7 cells, probably as a result of loss of sensitivity to the compounds. Noteworthy are two iridium complexes (3 and 6) that were non-cytotoxic and evoked an anti-inflammatory response toward LPS-induced NO production, related to the structure of these compounds. The lack of cytotoxicity for these iridium complexes may be associated with higher indifference and slower kinetics, compared to Ru and Os analogues. Their anti-inflammatory activity comes from the configuration with a deficiency of electrons of the metal which enables binding of the NO ligand with the metal ion. Strong anti-inflammatory action of non-cytotoxic complexes 16-e can become useful in the treatment of diseases, reducing inflammation [4].

The authors emphasized the importance of two iridium compounds (Ir (η5-pentamethylcyclopentadien) (1,2-dicarb-klozo-dodecarboran-1,2-ditiolato)) [3] and (Ir (η5-pentamethylcyclopentadiene) (benzene-1,2-ditiolato)) [6] which were non-cytotoxic and induced a full anti-inflammatory response towards LPS-induced NO [4]. It is worth noting that evidence from literature showed that NO is associated with IBD and that it is linked to disease activity [50]. Zhang et al. claim that the configuration with the deficiency of electrons in the center of the metal enables the binding of the NO ligand with the metal ion. The obtained results thus warrant the design of organometallics with other metals in active centers to bind NO and decrease the development of inflammation [4].

## 3. Antibacterial Compounds

Inflammatory Bowel Diseases are associated with an imbalance in the gut between pathogenic microorganisms, which can proliferate on the epithelial surface and then attack the mucous membrane, and commensal microorganisms, which prevent gut colonization by pathogens [51,52]. Currently, it is suggested that many bacteria have pathogenic effect on intestinal function, whereas *Escherichia coli* is the first characterized bacterium undoubtfully associated with the development of IBD. In line, numerous studies show that *E. coli* occurs at elevated numbers in tissues of patients with IBD [53,54,55]. Mylonaki et al. [56] showed that rectal biopsies of UC patients contained high colonization of *E. coli* as well as *Clostridium* compared to a healthy control group [56]. Complexes also show antibacterial activity with potential anti-inflammatory effects. For example, Souvik et al. [57] designed four mononuclear complexes [{PO2(NH2MePy)2}2M) (M = Zn [1], Cu [2], Co [3], Ni [4]], which were subsequently characterized in vitro by the agar diffusion method. The results indicate that complexes 2–4 show significant cytotoxicity towards *E. coli*. [57].

### 3.1. Gold

**Figure 6:**
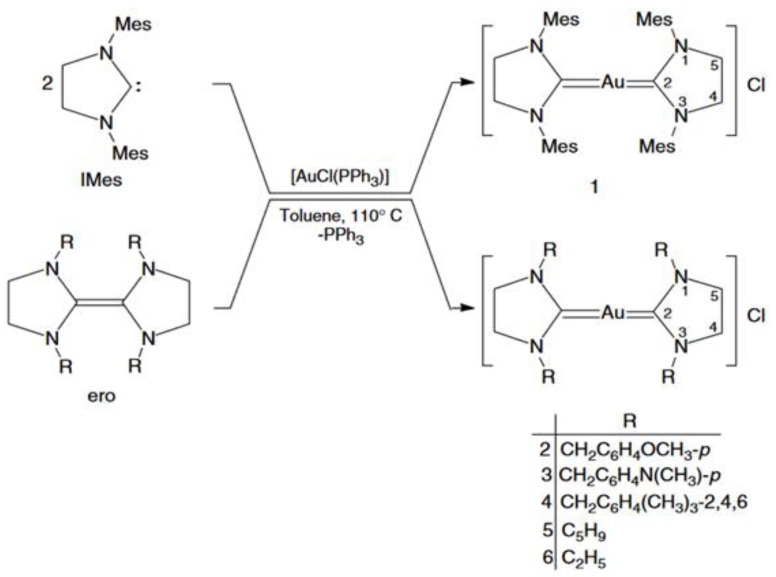
Synthesis of gold(I)-NHC complexes used for studies [58].

An important issue that should be taken into account in IBD patients is bacterial infections. Ozdemir et al. [58] investigated the 3-diorganylimidazolidin-2-ylidene (NHC) gold (I) complexes as potential antimicrobial agents (Figure 6). Complexes 1–3 showed remarkably selective and effective action against Gram-positive and Gram-negative bacteria. For example, the p-methoxybenzyl derivative (complex 2) inhibited the growth of *Pseudomonas aeruginosa*, *Staphylococcus epidermidis*, *Staphylococcus aureus* and *Enterococcus faecalis* with minimum inhibitory concentration (MIC) values of 3.12, 6.25, 3.12 and 3.12 μg/mL respectively. The authors found that the intensity of antimicrobial activity is strongly related to the rate of ligand exchange of gold (I) complexes [58].

### 3.2. Zinc

**Figure 7:**
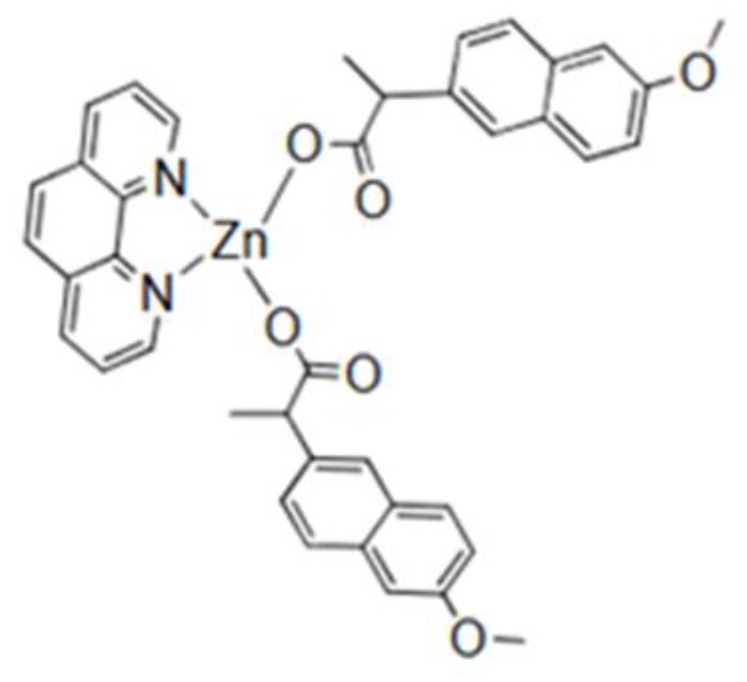
Zinc naproxen complex exhibiting strong anti-gram-positive and anti-gram-negative bacteria properties [60].

**Figure 8:**
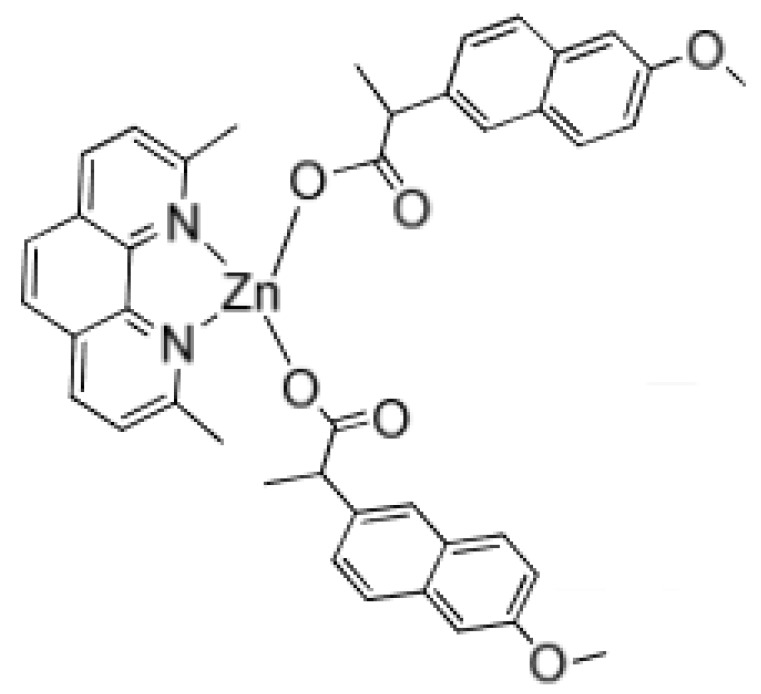
Zinc naproxen complex exhibiting strong antimicrobial properties against *S. aureus* [60].

Noteworthy, zinc is an important element playing a crucial role in several processes in the living organisms. Zinc ions form complexes with proteins, nucleic acids and participate in metabolism, regulation of genetic information expression storage, synthesis and action of peptide hormones [59]. Zinc may also inhibit the growth of *Staphylococcus aureus* [60] which occasionally occurs during the course of IBD [61]. Interestingly, it was evidenced that zinc and naproxen may form a complex (Figure 7) which exhibits strong anti-Gram-positive and anti-gram-negative bacteria properties (both *S. aureus* and *E. coli*). Another zinc compound (Figure 8), resulting from the modification of the previous compound, has a greater effect on S. aureus than on Gram-negative bacteria [60].

In addition, it has been shown that the hetero-tri-organometallic compound consisting of a nucleic acid backbone with ferrocene (FcPNA) also displays bactericidal action against Gram-positive bacteria, including *S. aureus*. FcPNA belongs to a new class of synthetic antibiotics with a completely new structure whose action consists in integration with the lipid layer and changing the architecture of the membrane. FcPNA is a promising compound because it has a strong antibacterial activity and low cytotoxicity [62].

### 3.3. Triphenyltin Complex (LTP)

The structure that attracted the most attention, triphenyltin complex (LTP) displayed strong antibacterial properties against *S. typhi* and *S. aureus* [63], which cause infectious colitis, associated with overexpression of TNF-α, IL-1β, and IL-6 [64]. The tributyltin complex (LTB) has also shown excellent activity against *S. aureus* species, reaching a value of MIC = 8 μg/mL and against *Shigella spp* and *S. typhi*. Of note, selected other synthesized complexes also showed antibacterial properties by acting on the same strains of bacteria [63].

### 3.4. Free Radicals Related in IBD

Free radicals play an important role in the development of inflammatory diseases. The natural consequence of oxygen metabolism is the formation of reactive oxygen species (ROS) and reactive nitrogen species (RNS). In low concentrations, they can protect against pathogens and are responsible for protein phosphorylation or cellular immunity [65], while high concentrations of these molecules dysregulate the inflammatory response of the body, disturb the protein and lipid composition or cause changes in DNA [66]. The development of IBD is associated with an imbalance between ROS and antioxidant activity, causing increased ROS production and leading to oxidative stress (OS). ROS and RNS induce cytokine secretion such as IL-4, TNF-α, IL-1β, IL-6 and IL-8, which play an important role in IBD. Consequently, excessive production of ROS/RNS is the cause of prolonged intestinal inflammation, while the body’s antioxidant defense can offset the negative effects of excessive ROS production. The antioxidant capacity of IBD patients is reduced, even in the asymptomatic phase of the disease [67]. Recent studies suggest that the administration of antioxidants with additional anti-inflammatory effects may be beneficial in the treatment of IBD [68]. Of note, metals are an important element in the protection of the gastrointestinal tract and most antioxidant enzymes are dependent Zn, Fe, Cu or Mn [69]. Synthetic compounds containing these metals may have similar functions and may be used for IBD treatment.

It is well established that iron complexes with dithiocarbonates or ruthenium compounds with polyamine/polycarboxylate scaffolds are effective as ROS scavengers [70,71]. Currently, double-acting compounds are being developed, such as molecules from Mn (II), which play the role of ROS scavengers and are also enzyme mimetics. [70].

**Figure 9:**
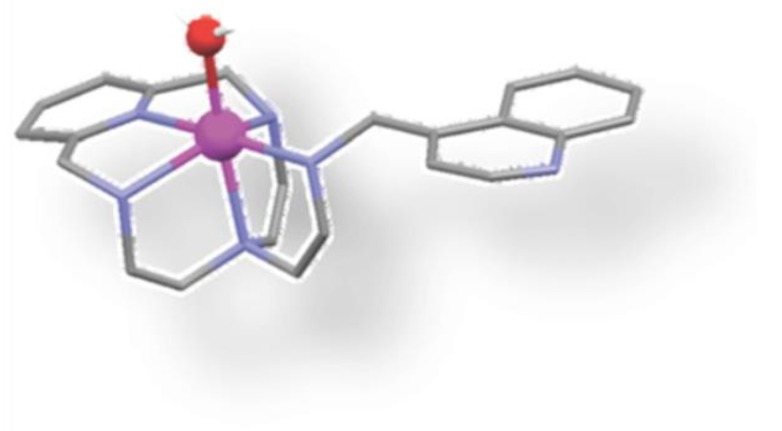
Stick and ball structure of Pytren4Q-Mn (Mn-L1). In red oxygen, pink manganese and blue nitrogen [72].

Superoxide dismutase (SOD) enzymes are responsible for deactivation of peroxide radicals $O_{2}^{-}$ and the impairment of this system is associated with pathogenesis of diseases associated with the ongoing inflammatory process [72]. Preliminary studies suggest that a SOD mimetic compound, the Mn^II^ complex of polyamines Pytren4Q (Mn-L1) (Figure 9) displays high antioxidant activity in bacteria and yeasts and thus may possess an anti-inflammatory effect [72]. Further investigations showed that Mn-L1 provided protection against LPS-induced proinflammatory effects in both human macrophages and mice. The use of Mn-L1 compound decreased the secretion of proteins (secreted as a result of induction by LPS), probably partly due to inactivation of the MAP kinase involved in the Act pathway modulating the level of proinflammatory cytokines in macrophages. In the mouse sepsis model, Mn-L1 significantly improved the survival of mice treated with a lethal dose of LPS [72]. Mice treated with Mn-L1 had lower levels of proinflammatory cytokines TNF-α and IL-6 compared to control mice injected with LPS alone, and that Mn-L1 decreased the activation of mouse hepatic macrophages induced by LPS [72].

**Figure 10:**
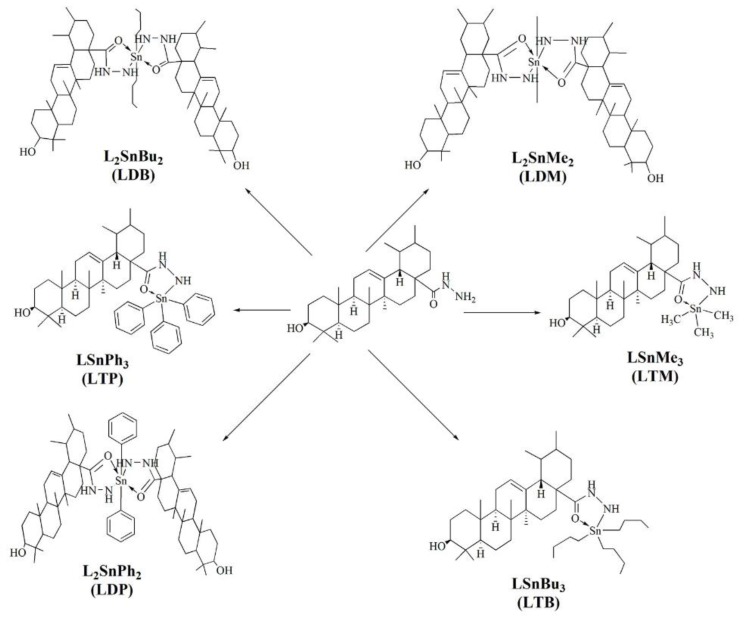
Synthesis of ursolic acid-tin complexes [63].

In the case of free radicals, another issue should be raised. Ursolic acid (UA) is a pentacyclic triterpenoid carboxylic acid and the main product of many plants, including apples, basil, cranberries, peppermint, oregano, which has antioxidant and anticancer properties [73]. Numerous reports indicate that the anticancer, anti-inflammatory and proapoptotic effects of UA are the result of its ability to inhibit the immunoregulatory transcriptional factor NF-κB [74,75]. Jabeen et al. [63] evidenced that UA complexes with metals (Figure 10) may show strong therapeutic properties. Newly designed compounds combined hydrazine, hydrazone and other analogues, which act principally as sweepers of free radicals and tin, which strongly affects the biochemical properties of metal complexes. The obtained organic-tin complexes (IV) displayed strong antifungal and antibacterial effect [63].

## 4. Carbon Monoxide Donors Related in IBD

Carbon monoxide (CO) is a physiological product of heme oxygenase produced during catabolic breakdown of heme, with antioxidant and antiapoptotic function. The administration of CO can thus serve to treat a group of diseases, such as IBD, which are induced by ROS [76]. Tricarbonyldichlororuthenium (II) dimer (CORM2) is widely used as a CO donor in various in vitro studies. However, CORM2 is characterized by low solubility in water and a very short half-life when releasing CO. Consequently, SMA (water-soluble, styrene-maleic acid copolymer)/CORM2 micelles were created for better performance and improvement of physicochemical properties. They are highly soluble in water and have a significantly extended circulation time. These molecules are selective towards inflammatory changes because they exploit the effect of increased permeability. Inflammatory tissues are characterized by increased vascular permeability due to overproduction of many vascular mediators; in effect, large drug molecules will accumulate in diseased sites, thus not penetrating the unchanged tissues [76].

**Figure 11:**
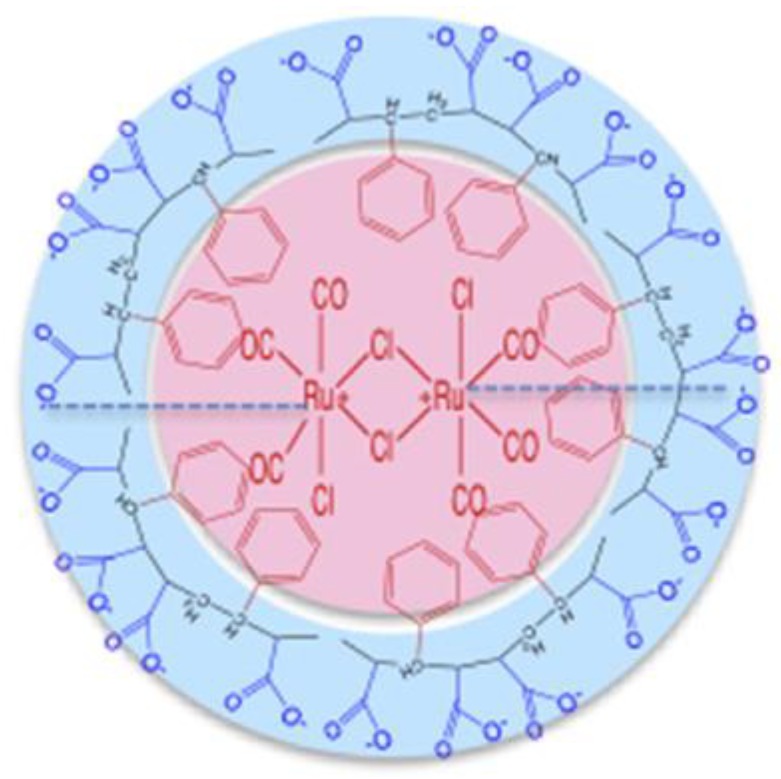
SMA/CORM2 micelle structure. In blue SMA formed a hydrophilic outer surface, in red CORM2 formed a hydrophobic core [76].

Studies have showed that the administration of free CORM2 induced a rapid increase in the level of CO (3.5-fold higher values) in the blood after 2 h. However, CO level rapidly decreased due to its removal by breathing. In the case of administration of SMA/CORM2 (Figure 11), the concentration of circulating CO increased slowly, in a linear, constant manner. It may be thus suggested that SMA/CORM2 can improve the bioavailability of CO in vivo compared to CORM2. Furthermore, in the mouse DSS model of colitis, SMA/CORM2 showed a significant protective effect on tissues by releasing CO from the micelles. This implies that SMA/CORM2 micelles can be used to treat ROS-related inflammatory diseases, including IBD [76].

## 5. Organometallic Compounds in CRC

Metal-based medicines play a key role in cancer treatment due to the high clinical success of platinum-based medicines. Currently, cisplatin, carboplatin and oxaliplatin are used in clinical treatment of a wide range of cancers [77]. However, as established platinum drugs have a number of serious drawbacks, such as limited spectrum of anticancer effects, strong systemic toxicity and frequent formation of chemical resistance, intensive research efforts are currently being made to discover new metal compounds with more beneficial anticancer effects [78].

CRC is one of the most serious IBD complications and accounts for 10–15% of IBD deaths [79]. Patients suffering from IBD for 8–10 years have an increased risk of dysplasia and CRC. In the case of IBD, the production of various inflammatory cytokines increases due to the penetration of immune cells in the intestinal mucosa and the excessive production of inflammatory cytokines is associated with carcinogenesis [80].

Early detection of disturbing lesions is a crucial element in CRC prevention. Endoscopic biopsy is the standard in the diagnosis of CRC, however, in the case of UC it is not a fully reliable examination as dysplasia may develop in the proper-looking mucous membrane [81]. Therefore, new screening strategies that are more sensitive to high-risk patients are under development. Recently, a Cu-DOTA-cetuximab-F (ab’) 2 imaging probe was successfully delivered to noninvasively assess epidermal growth factor receptor (EGFR). This protein was chosen as the target of the new probe as it was able to detect the tumor even in colitis, had a high expression visible in the vast majority of CRC, and there was no overexpression in the normal colorectal mucosa and in inflammatory cells associated with chronic colitis. Cu-labelling allowed for a more accurate identification of lesions during the study and made it possible for the probe to have a suitable half-life time [81].

### 5.1. Ruthenium

In recent years, unique properties of organometallic complexes based on Ru have been identified, including the ability to form a large number of potential auxiliary and therapeutic molecules. Past results show that Ru-based organometallic compounds are promising candidates for cancer and metastatic drugs. Fernandes et al. [82] developed new organometallic compounds of ruthenium (II) with the general formula [(η5-C5H5) M (PP) Lc] [PF6], bearing carbohydrate derivative ligands (Lc). The potential effect on tumor cell viability was determined using HCT116 colorectal cancer cell line cells. Researchers investigated the effect of 23 compounds on cytotoxic activity and compared by analyzing the IC50 values. The IC50 values of two compounds, 14Ru (0.45 μM) and 18Ru (0.44 μM), were similar to those obtained using oxaliplatin (0.45 μM), which is a platinum chemotherapeutic drug used specifically for CRC. In addition, 14Ru causes dose-dependent death of HCT116 cells, causing an increase in caspase-3 and -7 activity levels and modulates apoptosis in a dose-dependent manner. 14Ru induced a dose-dependent increase in HCT116 apoptosis, leading to an increase from 16 to 22 and 43% of apoptotic cells at 1 and 2 µM concentrations, respectively, whereas compared to vehicle control exposure (*p* < 0.01). The authors also emphasized that 14Ru intensifies apoptosis compared to oxaliplatin at 2 μM (*p* < 0.01), which caused apoptosis of 30% of HCT116 cells (*p* < 0.01) [82].

**Figure 12:**
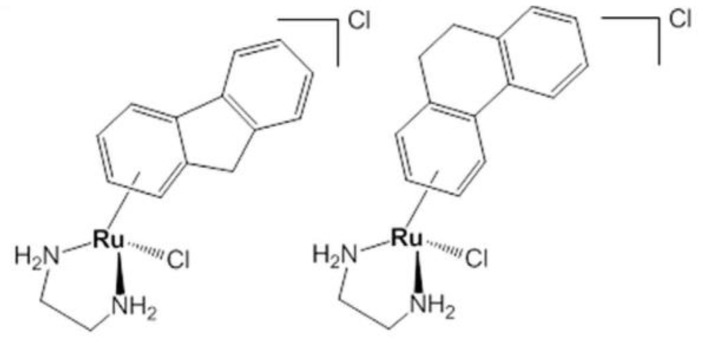
Chemical structures of AH54 (left) and AH63 (right) [83].

The greatest improvement in clinical outcomes in patients with solid tumors is due to simultaneous treatment with chemotherapy and radiotherapy (RT), which induces the death of cancer cells by DNA damage. Carter et al. [83] believed that ruthenium-arene complexes are radiation-sensitive agents when used in combination with RT, increasing the effectiveness of cancer treatment. The authors designed ruthenium complexes with cytotoxic activity. Among those, two compounds were developed that were showed to display facial chirality, related to different orientations in which the ligand arene can be associated with the core of ruthenium (AH54 and AH63) (Figure 12). The effect of p53 status on the response to AH54 and AH63 was tested using isogenic HCT116 cells, which were of wild p53 (+/+), p53 null (−/−) or mutated p53 (S248W/−) type and carried a common tumor mutation into the DNA binding domain of p53 protein. The subject compounds displayed radiosensitizing activity in p53-wildtype cells compared with p53-null or p53-mutated, and a mechanism of their action has been described that appears to involve DNA damage. Among the population of wild type cells, significant cell cycle inhibition in the G2/M phase was demonstrated. However, mutant p53 cells showed little effect and cells devoid of p53 replicated their DNA in a normal way. Apoptosis results are encouraging because apoptosis occurred in both HCT116 cells of wild p53 and p53 mutated type, which indicates that these drugs may be effective in tumors where p53 has been mutated. In addition, it was found that cytotoxicity increased six-fold when the phenyl group was included in the para position with respect to the bound arena [83]. Confirmation of these results in in vitro studies would allow the development of new anti-cancer agents using Ru by combining with RT.

**Figure 13:**
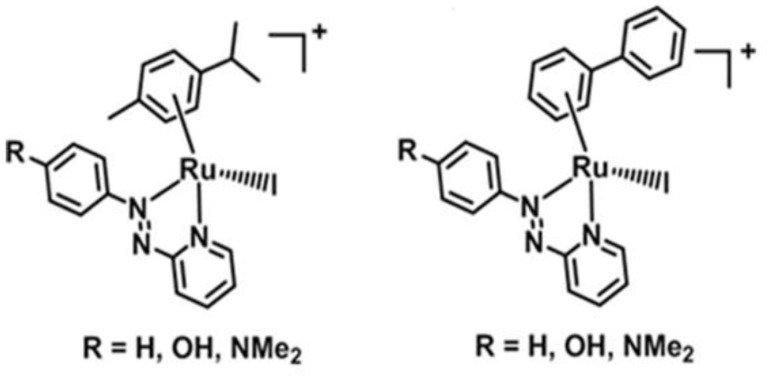
Structures of ruthenium complexes synthesized in this work [84].

Dougan et al. [84] discovered that the ligand chelating in the Ru arene complexes can have a huge impact on reactivity (Figure 13). Replacement of diamine with phenylazoazopyridine derivatives led to inert complexes with redox mechanisms of anticancer activity [84].

Currently, several Ru complexes are in phase I or II clinical trials [85,86]. Based on structure-activity studies, Ru complexes can function to inhibit cancer cells through mechanisms similar to those of cisplatin [87]. Moreover, other organometallic complexes of ruthenium (II) arena, RM175 and RAPTA were also promising [88].

### 5.2. Iridium

Design of organometallic iridium compounds as potential therapeutic agents is still underdeveloped, partly caused by the neutral character of the low spin d6-metal ion. Lord et al. [89] described a new library of organometallic compounds of iridium (III) type Cp*IrCl(N,O) Cp* = pentamethylcyclopentadienyl and N,O=a functionalized β-ketoiminato ligand) showing cytotoxicity to several cancer cell lines including colorectal cancer line. The authors presented the results of their research as selectivity coefficients (SR), which are defined as the ratio of the mean IC50 for normal cells divided by the mean IC50 for each line of cancer cells studied. The most significant effects were observed for compounds 4 and 9, which can be described as selective against cancer, having SR > 1 which indicates a preferential selectivity over cancer cells compared to normal cells. In order to evaluate selectivity against cancer cells, compounds were screened for normal prostate cells (PNT2) and abnormal retinal epithelial cells (ARPE-19). The values of SRs in relation to HCT119 p53 −/− for 4 and 9 were 14.07 and 3.97 (PNT2) and 35.59 and 20.28 (ARPE-19) respectively. Comparing the above results with SF values for cisplatin of 0.82 and 0.74, the authors indicated that these compounds are non-toxic to normal cell types, as opposed to cisplatin, which remains cytotoxic. In addition, studies using the HCT116 cell line, in particular HCT116 p53 −/−, showed a sensitivity index (SF) of 26 (compared to cisplatin SF = 0.43).

### 5.3. Gold Complexes and Anti—Thioredoxin Reductase Activity

Thioredoxin reductase 1 (TR1), a protein belonging to the selenoprotein group, plays a role in both preventing and promoting cancer [90]. TR1 is one of the main regulators of the redox reaction and is involved in cell proliferation, angiogenesis, and DNA repair [91]. Zhang et al. [21] described Au (I)-NHC complexes as strong TrxR inhibitors, whose action is based on increased ROS formation, induced apoptosis and strongly influenced cellular metabolism. The examination of the complexes [Au (NHC) L] (L = Cl, NHC or PR3) using atomic absorption spectrometry (AAS) revealed that the binding reactions with serum albumin were sequential: Au (NHC) Cl] > [Au (NHC) (PR3)] I > [Au (NHC) 2] I. The activity of TrxR inhibition of gold complexes increased in the same order [21].

### 5.4. Osmium Complexes

**Figure 14:**
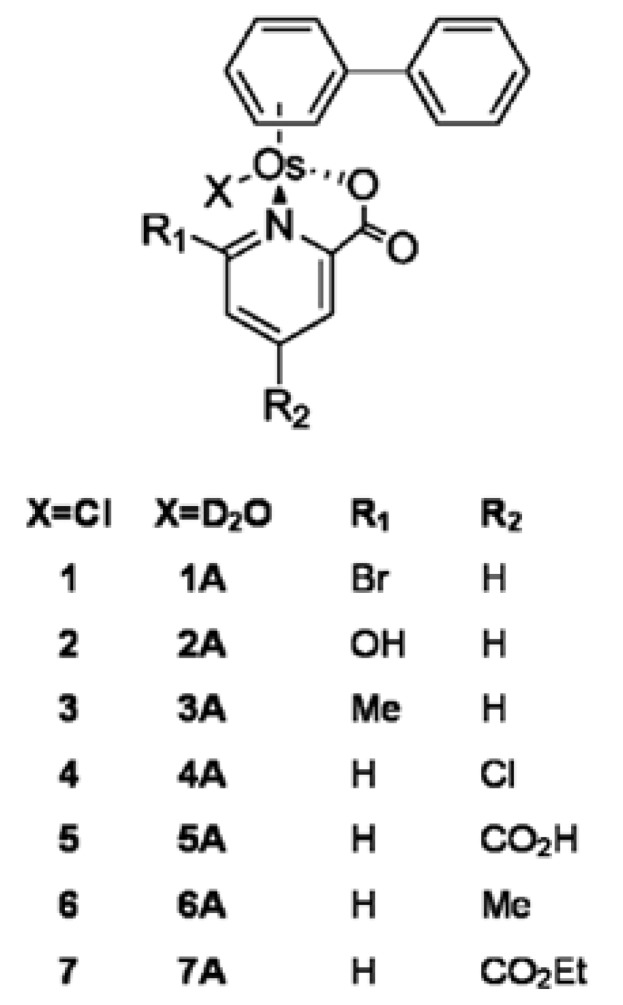
Osmium complexes studied in this work [92].

Rijt et al. [92] synthesized the chlorido osmium(II) arene complexes [(η6-biphenyl)OsII(X-pico)Cl] containing X = Br, OH, and Me as ortho, or X = Cl, CO_2_H, and Me as para substituents (Figure 14). The authors determined their cytotoxicity against HCT116, but the complexes were also tested in other tumors such as lung cancer A549, ovarian cancer A2780 and cisplatin-resistant ovarian cancer A2780cis. Complexes 1, 2, 3 and 5 were non-toxic at the highest concentration of 50 μM in all four cell lines. P-Cl and p-Me analogs (4 and 6, respectively) showed promising toxicity similar to that found for cisplatin in human A2780 ovarian cancer and HCT116 colon cancer cell lines. An important result was that compounds 4 and 6 overcome cisplatin resistance in A2780cis cell line [92].

**Figure 15:**
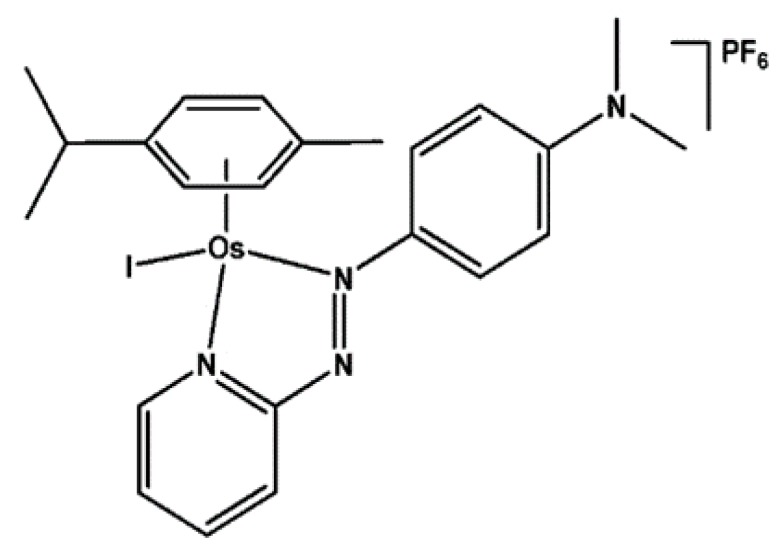
The structure of osmium(II) complex [93].

Fu et al. [93] demonstrated that the azopyridine complex OsII [Os (h6-p-cym) (azpy-NMe2) I] PF6 (Figure 15) is active at concentrations below micromolar in the HCT-116 colon cancer cell line and in other cancer lines, for example, lung cancer A549 or prostate cancer PC-3. Notably, in case of colorectal cancer line the IC50 value was 220 nM [93]. In further studies it was found that the examined organometallic compound induces a delay in tumor growth in the HCT-116 model in comparison with untreated control (*p* < 0.01). Unfortunately, the distribution of the compound in plasma and tissues after in vivo administration in mice with heterotransplant containing HCT116 showed that osmium can be detected in the tumor and in all tissues. Moreover, the amount of osmium in plasma was low after 5 min, suggesting a large volume of distribution or a high level of distribution among tissues. Studies on plasma, tumor and normal tissue distribution suggest that it is possible to optimize therapeutic activity by using multiple dose regimens with no risk of toxicity beyond the purpose of the compound [94].

### 5.5. Platinum

The discovery of cisplatin by Barnett Rosenberg in 1960 was a huge advance in the history of metal-based compounds used in the treatment of cancer [95] which encouraged the design of several other platinum drugs, such as cisplatin, carboplatin and oxaliplatin. Platinum-based drugs are particularly active against germ-cell tumors. In addition, they play a major role in the treatment of colon cancer, head and neck or bladder cancer [96]. The mechanism of cisplatin action is based on its interaction with DNA, leading to DNA damage and cancer cell death; however, its use may cause neuro- and nephrotoxicity [97].

Oxaliplatin is a third-generation platinum-containing drug, which affects tumors resistant to cisplatin and carboplatin. It acts as DNA alkylating agent, crosslinking two adjacent guanine bases or adjacent guanine and adenine [98], causing inhibition of DNA synthesis. Oxaliplatin has a 1,2-diaminocyclohexane ligand (DACH) in its structure, which causes that DNA is more difficult to repair, thus improving the effectiveness of treatment [99]. Oxaliplatin has fewer side effects than cisplatin, but they still narrow down its therapeutic index [100]. Oxaliplatin is approved for the treatment of CRC and in therapy is combined with 5-fluorouracil (5-FU), leucovorin, folinic acid. The combination of these drugs is referred to as FOLFOX and is a first-line chemotherapy strategy for metastatic CRC.

## 6. Main Problems Associated with Metal Compounds

Synthesis of organometallic compounds and metal complexes is a multistep process and at each step several issues need to be solved. For example, some ligands’ structure allows competitive coordination ability, like in the case of methionine or methyonyl-containing peptides [101]. In this case, isolation of single crystals, or NMR (attachment) and tandem MS/MS mass spectrometric experiments are necessary in order to determine the coordination in the obtained molecule [102]. In addition, common problems are related to the process of crystallization of highly insoluble compounds. Experts point to the synthesis of molecules by diffusion as the solution to the problem. In some cases, organic ligand is soluble only in DMF or DMSO which should not be used because they form complexes with metal(s) of interest. It is then advisable to verify the strength and solubility of the ligand-metal complex, as these will allow in many cases the purification of the main product. Finally, problems with the adhesion of organic compounds on metal surfaces are reported. These are overcome by modification of the surface, e.g., through partial oxidation.

## 7. Conclusions and Future Perspective

Organometallic compounds and metal complexes containing zinc, silver, gold or rhodium can give basis to the design of new therapeutic options, targeting—among others—gastrointestinal inflammation and cancer. Critical evaluation of available literature strongly suggests that the future research should focus in particular on the mechanism of action of organometallic compounds and metal complexes, and principally those containing gold. So far, studies confirm the effectiveness of gold compounds in arthritis, but none of them indicates the mechanism of action. Broader studies are thus still fully justified in order to assess the potential anti-inflammatory, anticancer and toxic profiles of organometallic compounds and metal complexes, gold-based included.

## Figures and Tables

**Table 1:** Therapeutic application of selected organometallic compounds.

Organometallic Compound	Application in Medicine/Mechanism of Action	Ref.
Na_2_[Fe(CN)(NO)]	Anti-hypertension	[21]
Irridium (III) Cyclopentadienyl containing C, N-chelating ligands Pyridyl Ruthenium (II) arene	Anti-tumor properties Aqua-adduct formation	[22] [23]
Irridium (III) cyclopentadienyl containing C,N or N,N–chelating ligands	DNA fragmentation Inhibition of protein synthesis	[24]
Osmium(II) arene AFAP51 Multi-targeted organometallic ruthenium (II)-arene	Anti-tumor properties	[25] [26]
Iridium compounds [Ir (η5-pentamethylcyclopentadien) (1,2-dicarb-klozo-dodecarboran-1,2-ditiolato)]	Anti-inflammatory action	[4]

## Abbreviations

5-ASA	5-aminosalicylic acid
5-FU	5-fluorouracil
AAS	Atomic absorption spectrometry
AgNPs	Silver nanoparticles
AzZnSA	5-(p-sulfophenylazo-)-salicylidenealanine-zinc
CD	Crohn’s disease
CO	Carbon monoxide
CORM2	Tricarbonyldichlororuthenium (II) dimer
CRC	Colorectal cancer
DACH	1,2-diaminocyclohexane ligand
EGFR	Epidermal growth factor receptor
IBD	Inflammatory bowel diseases
IFN-γ	Interferon-gamma
IL-1	Interleukin-1
IL-4	Interleukin-4
IL-6	Interleukin-6
IL-8	Interleukin-8
iNOS	Inducible NO synthase
LPS	Lipopolysaccharide
LTB	Tributyltin complex
LTP	Triphenyltin complex
NAE	NEDD8-activating enzyme
NF-κB	Nuclear factor-κB
NO	Nitric oxide
OS	Oxidative stress
RNS	Reactive nitrogen species
ROS	Reactive oxygen species
RT	Radiotherapy
SASP	Salicylazosulfapyridine
SMA	Styrene-maleic acid
SOD	Superoxide dismutase
STAT3	Signal transducers and activators of transcription 3
TNF-α	Tumor necrosis factor-alpha
TrxR	Thioredoxin reductase
TR1	Thioredoxin reductase 1
UA	Ursolic acid
UC	Ulcerative colitis
